# Quantitative Ultrasound Characterization of Intensity-Dependent Changes in Muscle Tissue During Percutaneous Electrolysis

**DOI:** 10.3390/jcm14124064

**Published:** 2025-06-09

**Authors:** Miguel Malo-Urriés, Jacobo Rodríguez-Sanz, Sergio Borrella-Andrés, Izarbe Ríos-Asín, Isabel Albarova-Corral, Carlos López-de-Celis

**Affiliations:** 1 Healh Sciences Faculty, Department of Physiotry and Nursing, University of Zaragoza, 50009 Zaragoza, Spain; malom@unizar.es (M.M.-U.); sergiocai04@gmail.com (S.B.-A.); irios@unizar.es (I.R.-A.); ialbarova@unizar.es (I.A.-C.); 2 PhysiUZerapy Health Sciences Research Group, University of Zaragoza, 50009 Zaragoza, Spain; 3 Department of Medicine, Faculty of Medicine and Health Sciences, Universitat Internacional de Catalunya, Sant Cugat del Vallès, 08195 Barcelona, Spain; 4 ACTIUM Functional Anatomy Group, Faculty of Medicine and Health Sciences, Universitat Internacional de Catalunya, Sant Cugat del Vallès, 08195 Barcelona, Spain; carlesldc@uic.es; 5 Department of Physiotherapy, Faculty of Medicine and Health Sciences, Universitat Internacional de Catalunya, Sant Cugat del Vallès, 08195 Barcelona, Spain; 6 Study Group on Pathology of the Locomotor System in Primary Care (GEPALAP), Institut Universitari d’Investigació en Atenció Primària (IDIAP Jordi Gol), 08007 Barcelona, Spain

**Keywords:** percutaneous electrolysis, dosage, quantitative ultrasound, galvanic current, muscle

## Abstract

**Background/Objectives:** Percutaneous electrolysis is a physiotherapeutic technique based on the application of galvanic current to induce structural and biochemical changes in musculoskeletal tissues. Although widely used in tendinopathies, its application in muscle tissue, particularly regarding optimal dosage, remains poorly understood. This study aimed to evaluate the dose-dependent effects of galvanic current on cadaveric muscle tissue (medial gastrocnemius) using quantitative ultrasound analysis, and to identify objective biomarkers to guide dosage. **Methods:** An experimental model was employed, applying galvanic current at varying intensities (0–10.0 mA) to 29 samples of cadaveric medial gastrocnemius. Quantitative ultrasound parameters were measured, including geometric and textural features. Correlation analyses and simple and multiple linear regressions were performed to assess the relationship between current intensity and ultrasound variables. Additionally, dose segmentation into three groups (low: 0–1.0 mA, medium: 1.0–4.0 mA, high: >4.0 mA) allowed for comparative statistical analysis using Kruskal–Wallis and post hoc Mann–Whitney U tests. **Results:** Significant dose–response relationships were observed in key ultrasound parameters, including A_Number, A_Area, A_Perimeter, and A_Contrast (*p* < 0.001). Regression analysis revealed that a combination of A_Area, A_Number, and A_Perimeter accounted for 66.7% of the variance in applied dose (R^2^ = 0.667, *p* < 0.001), leading to the creation of the predictive variable Muscle_Electrolysis_Dose. Comparative analysis confirmed significant differences between low-, medium-, and high-dose groups, particularly between lower and higher doses. **Conclusions:** Quantitative ultrasound effectively detects structural changes in muscle tissue following percutaneous electrolysis. The results support the development of objective, image-based criteria for optimizing galvanic current dosage, enhancing the precision and personalization of treatment.

## 1. Introduction

Percutaneous electrolysis (PE) is an innovative intervention that delivers a direct current via a dry needling needle, aiming to trigger controlled electrochemical and biological alterations within the target tissue [1]. The underlying mechanism involves the electrolysis-driven breakdown of molecular structures through direct current, which also promotes the formation of reactive oxygen species and shifts in local pH balance, facilitating a localized inflammatory cascade that supports regenerative processes [2]. While tendon tissue has traditionally been the primary focus of PE [3,4,5,6], recent studies suggest that muscle tissue—particularly in the presence of injury—can also benefit from this intervention [7]. In acute muscle lesions, such as tears, PE may help modulate excessive inflammation and regulate the local immune response, creating a more favorable environment for healing [2]. Additionally, experimental studies have shown that the greater effectiveness of PE in muscle tissue is associated with the use of higher electric charges, indicating a dose-dependent biological effect [8]. Clinical evidence supports the application of ultrasound-guided PE in muscular injuries, especially in the soleus and gastrocnemius muscles, which are commonly affected in athletes. Two recent clinical trials in patients with chronic soleus injuries reported significant improvements in pain, function, and dorsiflexion range of motion when PE was combined with specific therapeutic exercise programs [9,10]. These results highlight the potential of PE to enhance recovery in muscular pathologies, particularly when guided by imaging and integrated into multimodal treatment approaches. Despite these promising findings, one of the main challenges in the clinical application of PE remains the definition of an optimal dose tailored to the tissue characteristics and individual response [11]. Current protocols often rely on standardized parameters such as intensity (mA) and duration (s), without considering the specific structural or physiological properties of muscle tissue. The commonly used model of dosage calculation (Q = I × t) stems from electrochemical systems and may not adequately reflect the biological complexity of human tissues [12]. Identical electric charges applied with different combinations of current and time may lead to different outcomes, especially in heterogeneous tissues like skeletal muscle [11]. Moreover, despite the increasing use of PE in clinical practice, particularly in the management of chronic tendinopathies, its application in muscle tissue remains largely empirical. Muscle displays distinct anatomical and physiological characteristics—including greater vascularity, different connective tissue composition, and higher metabolic turnover—that may influence its response to galvanic current. Yet, most existing protocols extrapolate dosing parameters from tendinous models without adjusting for these differences. Furthermore, clinical application typically relies on fixed settings, with limited feedback on the actual structural effect induced in the tissue. This lack of standardization, coupled with the absence of objective biomarkers to guide treatment, underscores the need for more precise and individualized dosing strategies in muscular applications. Since galvanic current induces visual changes in the tissue—such as hyperechoic foci attributed to the formation of hydrogen gas microbubbles [13]—ultrasound could provide a valuable real-time biomarker of physiological response.

In this context, we hypothesize that the structural changes visible on ultrasound after PE could serve as a criterion for dose optimization. Specifically, we aim to assess the effects of varying galvanic current intensities on cadaveric muscle tissue, using the medial gastrocnemius as a model. Through quantitative ultrasound analysis, this study seeks to identify dose-dependent tissue response patterns and determine if there is a physiological threshold beyond which additional current produces diminishing returns. This approach could lay the foundation for a more individualized and responsive dosing strategy in PE, guided by the tissue’s real-time reaction rather than fixed electrical parameters.

## 2. Materials and Methods

### 2.1. Study Design

An in vitro experimental study was conducted using cadaveric muscle tissue, with the medial gastrocnemius muscle serving as the anatomical model. The primary objective was to analyze the effects of PE on muscle structure under varying intensities of galvanic current, assessing changes through quantitative ultrasound imaging analysis.

This study was approved by the Local Ethics Committee of the Center (CBAS-2023-11), and all procedures adhered to the principles of good scientific practice in the handling of biological specimens to ensure data reliability and reproducibility.

A total of 29 samples of cadaveric medial gastrocnemius muscle were used (50% male, all of Caucasian ethnicity, with a mean age of 73.7 years (SD = 9.94)). All specimens had been stored at −20 °C and were acclimatized at room temperature for 48 h prior to the experimental procedures.

### 2.2. Experimental Protocol

Each sample received a randomly assigned dose of galvanic current, with 29 different intensities ranging from 0.00 mA to 10.00 mA. Initial intensity increments were set at 0.10 mA, increasing from 0.00 to 1.00 mA, followed by 0.50 mA increments up to the maximum intensity (10.00 mA). Each application lasted exactly one second.

**Figure 1:**
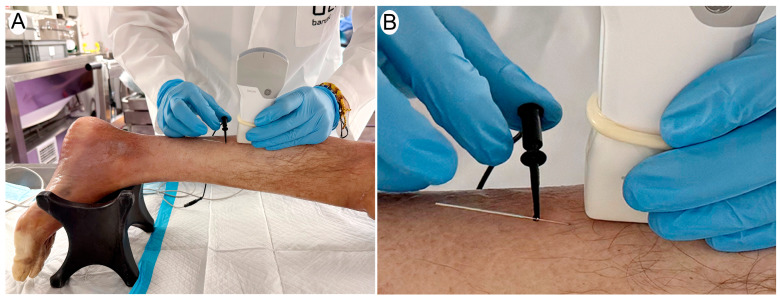
(**A**) Overview of the procedure showing the cadaveric limb positioned for ultrasound-guided percutaneous electrolysis. (**B**) Close-up of the needle insertion under ultrasound guidance using a longitudinal in-plane approach. A 40 mm × 0.30 mm needle is inserted into the medial gastrocnemius muscle, and galvanic current is applied using a bipolar system.

**Figure 2:**
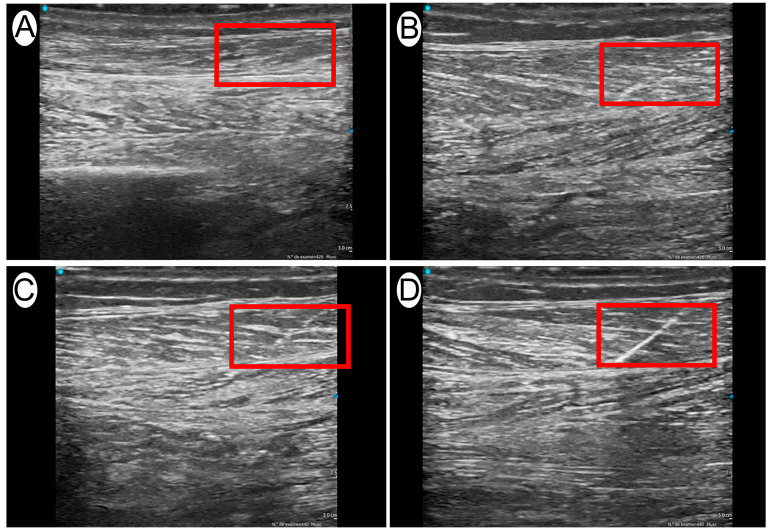
Representative ultrasound images of different PE doses applied for 1 s: (**A**) 0.10 mA; (**B**) 1.00 mA; (**C**) 2.00 mA; (**D**) 6.00 mA.

To ensure consistency, the medial gastrocnemius muscle was positioned in a standardized setup mimicking the typical clinical posture for intervention, with the knee slightly flexed to approximately 5° and the ankle in a neutral position (Figure 1). This positioning enabled homogeneous imaging conditions across all samples. The procedure was performed under ultrasound guidance using a longitudinal view of the medial gastrocnemius and an in-plane approach, with distal-to-proximal needle insertion. The galvanic current was applied by a specialist with over 10 years of experience in ultrasound-guided invasive techniques, specifically trained in PE. A 40 mm × 0.30 mm needle (Agupunt^®^, Barcelona, Spain) was used for all applications, and the current was delivered using the Bipolar System device, designed for controlled galvanic current application. Ultrasound evaluation was performed using a Vscan portable ultrasound system (General Electric, Boston, MA, USA) immediately after each dose was applied. Scanning was conducted in a longitudinal, in-plane view, selecting the highest quality image that best represented the treated area. Image acquisition was performed by an expert in musculoskeletal ultrasound with more than 15 years of clinical experience, ensuring accurate and reproducible assessments of structural changes. The acquired images were analyzed using UZ eDosifier, a custom adaptation of the UZ qTool software (V.1) specifically developed for the quantitative evaluation of electrolysis effects in muscle tissue [14]. This software enabled the objective extraction of metrics related to texture, echogenicity, and the internal structure of the muscle following galvanic current application (Figure 2). The quantified variables are presented in Table 1. This comprehensive analysis allowed for a quantitative assessment of the effects of PE in muscle tissue and facilitated the identification of response patterns and potential dosing thresholds.

### 2.3. Statistical Analysis

Statistical analysis was performed using IBM SPSS Statistics version 29.0. A multi-phase approach was adopted to assess the quantitative ultrasound changes following the application of different galvanic current intensities delivered via PE to the medial gastrocnemius muscle.

Initially, an exploratory analysis using scatter plots was conducted to examine the relationship between the applied dose and the ultrasound-derived variables. This analysis enabled the identification of general response patterns, definition of trends, and detection of potential thresholds in the effect of galvanic current on muscle tissue.

Subsequently, a correlation analysis was performed using Spearman’s rank correlation coefficient to assess the strength and direction of the relationship between galvanic current dose and each quantified variable. This step provided preliminary insights into structural changes associated with increasing current intensity. For variables showing significant associations with the applied dose, a simple linear regression analysis was conducted. Regression coefficients (B) were calculated to estimate the magnitude of change per unit increase in current intensity. Coefficients of determination (R^2^), F-values from ANOVA, *p*-values, and 95% confidence intervals for the regression coefficients were reported.

Additionally, a multiple linear regression model was developed to combine the most significant predictors into a single optimized summary variable. This new predicted variable integrated the contribution of the different ultrasound parameters into a unified metric, allowing for a comprehensive evaluation of the global effect of galvanic current. Variable selection was based on statistical significance in prior analyses, and model residuals were examined to confirm the goodness of fit.

Finally, based on patterns observed in scatter plots and correlation analysis, dose ranges were segmented into three groups—low, medium, and high—to explore possible physiological thresholds in tissue response. To compare differences among the three dose groups, the Kruskal–Wallis test was applied, as some variables did not meet the assumptions of normality and homoscedasticity. When significant differences were found, post hoc comparisons were performed using the Mann–Whitney U test with Bonferroni correction for multiple comparisons. Boxplots were generated for each significant variable to visualize the distribution across dose groups and to graphically illustrate the differences found in the statistical analysis.

## 3. Results

### 3.1. Exploratory Analysis Using Scatter Plots

The exploratory analysis of scatter plots allowed for the identification of response patterns between the galvanic current dose and the quantified ultrasound parameters. It was observed that some variables displayed a clear dose-dependent response, while others exhibited high dispersion without an evident trend.

#### 3.1.1. Variables Showing an Apparent Relationship with Current Dose

**Figure 3:**
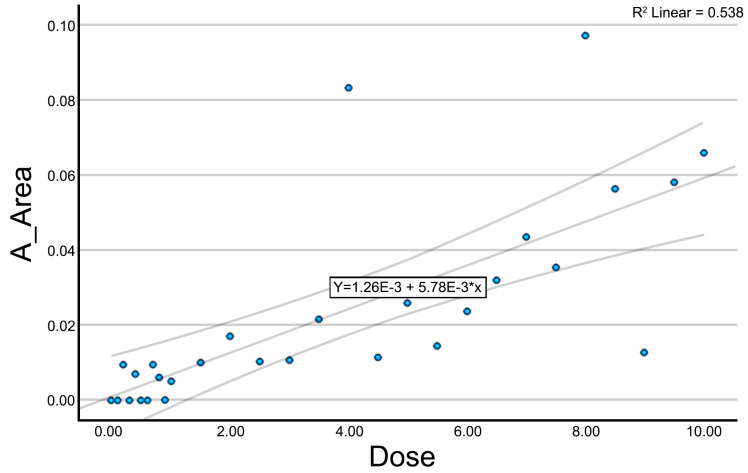
Scatter plot with trendline for the variable A_Area.

The variables A_Number, A_Area (Figure 3), and A_Perimeter showed a clear positive correlation with the applied dose. At low doses (<2 mA), values remained low or close to zero, whereas a progressive increase was observed beyond this threshold. At doses above 6.00 mA, values tended to stabilize, suggesting a potential saturation effect. Figure 3 illustrates this trend for the variable A_Area, where a distinct upward trajectory can be appreciated.

The variables A_Convexity and A_Homogeneity exhibited an inverse relationship with dose. At low doses (0–2 mA), values remained close to 1.00; however, a progressive decrease was observed beyond 2 mA, indicating a loss of homogeneity and convexity in the ultrasound image as current intensity increased. The variable A_ASM also showed a decreasing trend, albeit with a gentler slope and greater variability.

A_Contrast demonstrated a progressive increase with dose, with greater dispersion between 4 and 7 mA and a tendency toward stabilization at doses above 8 mA. Among the texture variables, some meaningful associations were identified. B_GLCM_SoSVariance and B_GLCM_Correlation showed a positive relationship with the applied dose, with more clustered distributions emerging beyond 4.00 mA. In contrast, B_GLCM_DVariance exhibited an inverse relationship with current intensity, showing a progressive decline in values.

The variables B_GLDS_Homogeneity, B_GLDS_Contrast, B_GLDS_ASM, B_GLDS_Entropy, and B_GLDS_Mean demonstrated significant correlations, although their values were notably dispersed, especially at low doses (<4.00 mA). From intermediate doses onward, values tended to cluster more tightly, suggesting a more distinct tissue response.

Specifically, B_GLDS_Homogeneity and B_GLDS_Mean showed a positive relationship, while B_GLDS_Entropy presented a negative association with the dose.

#### 3.1.2. Variables Without an Apparent Relationship with Dose

In contrast, other variables such as B_GLCM_Contrast, B_GLCM_SumAverage, B_GLCM_IDMoment, B_haar_mean, and B_haar_variance did not show a clear relationship with the applied dose. Their values exhibited high dispersion, without a consistent pattern indicating a dose–response dependency.

### 3.2. Correlation Analysis

A Spearman correlation analysis was conducted between the galvanic current dose and the various quantitative ultrasound parameters (Table 2). The results revealed several statistically significant correlations, suggesting potential associations between the applied intensity and the observed changes in muscular tissue.

Significant positive correlations (*p* < 0.01) were found between the galvanic current dose and the variables A_Number (ρ = 0.762), A_Area (ρ = 0.877), A_Perimeter (ρ = 0.873), and A_Contrast (ρ = 0.794) (Table 2). Likewise, a strong negative correlation was identified between the dose and A_Homogeneity (ρ = −0.828, *p* < 0.01), indicating a decrease in echogenic homogeneity with an increasing applied dose. Similarly, A_Convexity showed a significant negative correlation (ρ = −0.696, *p* < 0.01), while B_GLCM_Correlation (ρ = 0.558, *p* = 0.002) and B_GLCM_SoSVariance (ρ = 0.470, *p* = 0.010) also exhibited moderate positive correlations with dose (Table 2).

Among the texture-based variables, significant correlations were found for B_GLDS_Homogeneity (ρ = 0.379, *p* = 0.043), B_GLDS_Contrast (ρ = 0.368, *p* = 0.050), B_GLDS_ASM (ρ = 0.368, *p* = 0.049), B_GLDS_Entropy (ρ = −0.383, *p* = 0.040), and B_GLDS_Mean (ρ = 0.394, *p* = 0.035). In contrast, variables such as A_ASM (ρ = −0.101, *p* = 0.602), B_GLCM_Contrast (ρ = 0.173, *p* = 0.370), B_GLCM_SumAverage (ρ = 0.245, *p* = 0.201), and B_haar_mean (ρ = 0.243, *p* = 0.203) did not show statistically significant correlations with the applied dose (*p* > 0.05).

### 3.3. Simple Linear Regression Analysis

To assess the relationship between galvanic current dose and the ultrasound variables analyzed, simple linear regression analyses were conducted. Table 3 presents R^2^, standard errors of the estimate, B, and their 95% confidence intervals. A high R^2^ value indicated the proportion of variability in each dependent variable explained by the applied current dose.

The results revealed significant associations between galvanic current dose and several ultrasound variables, particularly A_Number, A_Area, A_Perimeter, A_Convexity, A_Homogeneity, A_Contrast, B_GLCM_SoSVariance, B_GLCM_DVariance, and B_GLCM_Correlation, all with statistically significant regression coefficients. Positive associations were observed for A_Number, A_Area, A_Perimeter, A_Contrast, and B_GLCM_SoSVariance, indicating a progressive increase in the number and size of the detected ultrasound structures and greater textural variability with higher doses. In contrast, A_Convexity and A_Homogeneity showed inverse relationships, suggesting that higher doses tend to reduce the convexity and homogeneity of muscular structures. Additionally, variables such as B_GLCM_DVariance and B_GLCM_Correlation also presented significant relationships, suggesting changes in the overall tissue texture.

### 3.4. Multiple Regression Analysis

A multiple linear regression analysis was conducted to identify the most relevant predictive variables explaining the galvanic current dose applied to the muscle tissue. All initial variables were included in the model, and the backward elimination method (as implemented in SPSS) was used to progressively exclude non-significant predictors. The final model included three predictors: A_Area, A_Number, and A_Perimeter (Table 4). The variable A_Area showed a statistically significant negative relationship with the applied dose (*p* = 0.009), indicating that larger area values were associated with lower doses of galvanic current. Similarly, A_Number also demonstrated a significant negative association (*p* = 0.031), suggesting that an increased number of structures corresponded to a reduction in the applied dose. In contrast, A_Perimeter exhibited a significant positive relationship (*p* = 0.002), with higher perimeter values corresponding to higher doses of galvanic current. The model demonstrated a multiple correlation coefficient of R = 0.816 and explained 66.7% of the variance in the applied dose (R^2^ = 0.667, adjusted R^2^ = 0.614), with a statistically significant *p*-value < 0.001. Based on this model, a new variable was generated, termed Muscle_Electrolysis_Dose, which integrated the information from the selected predictors. The residual analysis revealed an acceptable distribution with no systematic bias. The resulting model was robust and clinically interpretable.

**Figure 4:**
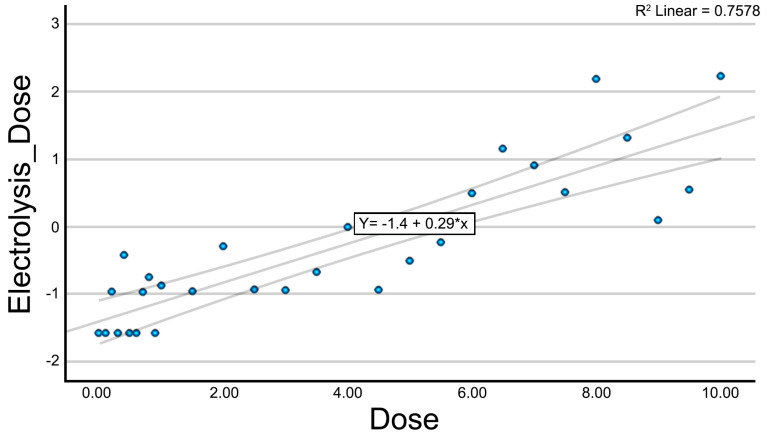
Scatter plot with the trendline for the variable Electrolysis_Dose.

The relationship between the new variable (Electrolysis_Dose) and the applied dose is shown in Figure 4. A consistent upward trend can be observed, indicating that the composite variable continues to reflect a clear dose–response relationship.

### 3.5. Dose Range Segmentation and Comparative Analysis

In order to assess the effects of galvanic current dose on muscle tissue, the data were segmented into dose ranges based on patterns observed in scatter plots and the distribution of the dependent variables. Therefore, three primary dose ranges were established:

- Low-Dose Group: 0–1.00 mA. This group included doses where minimal or no changes were observed in the analyzed variables.
- Medium-Dose Group: 1.00–4.00 mA. In this interval, the variables showed a progressive increase, suggesting a clear dose–response relationship.
- High-Dose Group: >4.00 mA. Beyond this threshold, several variables tended to stabilize or exhibited greater dispersion, which may have indicated a saturation or plateau effect in the tissue response.

This muscle-specific segmentation allowed for a more precise evaluation of potential physiological thresholds and intergroup differences. To this end, the Kruskal–Wallis test was used to compare the three dose ranges for each selected variable. When statistically significant differences were found, post hoc comparisons were performed using the Mann–Whitney U test with Bonferroni correction to identify specific pairwise group differences.

The results of this analysis are detailed in Table 5, which presents both the global Kruskal–Wallis statistics and the pairwise comparisons across the dose groups. This methodological approach revealed that muscle tissue response was clearly sensitive to the applied dose, particularly between the low- and medium-dose groups, and tended to stabilize at doses exceeding 4 mA.

### 3.6. Variable; Total N; Test Statistic; Degrees of Freedom; Asymptotic Significance (Two-Tailed); Low Vs. Medium Comparison (p); Low Vs. High Comparison (p); Medium Vs. High Comparison (p)

All analyzed variables showed statistically significant differences across the defined dose segments (*p* < 0.05), indicating that the applied galvanic current dose had a meaningful impact on the assessed muscular characteristics. The Kruskal–Wallis test confirmed the overall differences in all variables, with *p*-values < 0.001 in most cases.

Post hoc analysis using the Mann–Whitney U test with Bonferroni correction revealed that the main differences occurred between the low-dose group (0–1 mA) and both the medium- (1–4 mA) and high-dose groups (>4 mA). Specifically, variables such as A_Number, A_Area, A_Perimeter, A_Homogeneity, A_Contrast, and the derived variable UZ_eDosis showed statistically significant differences in these comparisons (*p* < 0.01). However, most variables did not display significant differences between the medium- and high-dose groups, suggesting a potential plateau in the tissue response from intermediate doses onward.

The variable UZ_eDosis, derived from the multiple regression model, also demonstrated significant differences among the three dose segments (*p* < 0.001), supporting its utility as a synthetic indicator of the global muscular response to galvanic stimulation.

## 4. Discussion

Establishing an appropriate dosage for PE continues to pose a considerable challenge in clinical practice, particularly due to the heterogeneous nature of tissue responses. Although PE is widely used in the treatment of musculoskeletal conditions, most protocols rely on fixed electrical parameters—such as current intensity and application time—without adequately accounting for the biological variability and specific properties of the target tissue. This limitation has been consistently highlighted in several systematic reviews, which emphasize the absence of a consensus and the lack of robust, evidence-based guidelines for determining optimal dosing parameters [15,16]. In the present study, we aimed to address this gap by evaluating the effect of graded intensities of galvanic current on cadaveric muscle tissue, specifically the medial gastrocnemius, using standardized quantitative ultrasound to identify dose–response patterns.

Our findings revealed that several ultrasound-derived parameters exhibited significant changes in response to increasing current intensity, supporting the hypothesis that quantitative ultrasound imaging may serve as an objective biomarker for guiding PE dosage in muscles. Key structural variables such as A_Number, A_Area, and A_Perimeter showed a clear dose-dependent increase, suggesting cumulative structural disruption with higher intensities. Conversely, A_Homogeneity and A_Convexity decreased with higher doses, indicating the progressive loss of tissue uniformity and geometric integrity. These patterns reflect the physiological mechanism of PE, which induces controlled microdisruption that may trigger regenerative cascades. Interestingly, the contrast parameter (A_Contrast) also increased consistently with intensity, possibly reflecting the formation of gas microbubbles and changes in echogenic heterogeneity, consistent with prior reports on electrochemical gas formation during electrolysis. Our findings revealed that several ultrasound-derived parameters exhibited significant changes in response to increasing galvanic current intensity, reinforcing the hypothesis that quantitative ultrasound imaging may serve as an objective biomarker for guiding PE dosage in muscle tissue.

These findings are consistent with previous in vivo studies, which have shown that low-intensity protocols, despite delivering equivalent electric charge, often fail to elicit detectable structural changes [12]. In our data, the 1–4 mA range appeared to be the most responsive window, with progressive and consistent changes observed in variables such as A_Number, A_Area, A_Perimeter, and A_Homogeneity. This supports the concept of a therapeutic dose window in which moderate intensities are sufficient to trigger key biological mechanisms like inflammation and apoptosis [2,11], both of which are essential for muscle tissue regeneration. Notably, values appeared to plateau beyond 4–6 mA, showing increased variability without further linear progression. Rather than indicating a true saturation of the tissue’s biological response, we hypothesize that this plateau effect may have stemmed from the accumulation of hydrogen gas and sodium hydroxide at the needle tip, as previously described in the literature [8,11]. This gas formation likely contributed to posterior acoustic shadowing, as observed in our experiment, where echogenic artifacts emerging from superficial gas pockets masked deeper structural changes. The consistent increase in A_Contrast at higher doses may have further reflected these electrochemical gas effects and alterations in local echogenic heterogeneity.

A novel and clinically valuable aspect of this study was the creation of the Muscle_Electrolysis_Dose variable—a composite score derived from A_Area, A_Number, and A_Perimeter through multiple regression modeling. This index explained 66.7% of the variance in applied dose (R^2^ = 0.667, *p* < 0.001), making it a promising quantitative marker of the structural impact of PE on muscle tissue. Similarly to our prior findings in tendinous tissue, this composite variable showed a consistent upward trend with increasing dose, offering an integrated and more stable representation of treatment effects. Its use in future real-time biofeedback systems could help personalize dosage based on measurable tissue response, rather than arbitrary intensity–time parameters. The final selection of A_Number, A_Area, A_Perimeter, and A_Contrast as key indicators was grounded in their consistent statistical performance and interpretability across multiple analyses. These parameters not only reflected the spatial extent of the induced structural alteration but also captured relevant echotextural changes associated with galvanic current effects. Although other potentially informative metrics—such as entropy or echogenicity—were initially considered, their greater variability and limited predictive power led to their exclusion from the final model.

To better define physiological thresholds in tissue response, we segmented the data into three dosage ranges: low (0–1 mA), medium (1–4 mA), and high (>4 mA). The Kruskal–Wallis and post hoc Mann–Whitney U tests confirmed statistically significant differences across all three groups for most variables, with the largest differences occurring between the low and medium ranges. A plateau effect was observed in several parameters above 4 mA, which may have reflected either a biological saturation in tissue response or a technical limitation due to ultrasound shadowing caused by accumulated electrolytic gas.

These findings are consistent with experimental studies demonstrating that the therapeutic effect of PE is not solely determined by the total electric charge (I × t), but rather by surpassing a minimum intensity threshold capable of activating key biological processes such as local inflammation, apoptosis, and tissue remodeling. Similarly to our study, the nonlinear dose–response curve we observed—characterized by minimal effects below 1 mA, a clear progressive response between 1 and 4 mA, and a plateau or saturation beyond 4 mA—mirrors previous in vitro findings showing that interleukin-1 beta (IL-1β) release by macrophages increases up to an optimal electric charge, beyond which cytotoxicity rises and regenerative signaling may diminish [11]. These findings emphasize that not only the cumulative dose, but also the specific intensity–time configuration determine the tissue’s biological response, a concept reinforced in both macrophage and muscle models [17]. From a translational perspective, this supports the clinical observation that intensities above a certain threshold (e.g., >1 mA in muscle) may be required to elicit therapeutic effects, particularly in chronic lesions where baseline inflammation is low and regenerative activity is limited [2]. Conversely, low-intensity protocols, despite delivering the same total electric charge, often fail to induce meaningful structural changes, as confirmed in our results and prior clinical data [12]. Overall, our study offered experimental validation for the hypothesis that galvanic current intensity plays a pivotal role in modulating PE’s impact, and that an optimal physiological window likely exists—between 1 and 4 mA in muscle—where regenerative potential is maximized while minimizing adverse effects such as excessive disruption or imaging artifacts [15]. These insights may inform future guidelines on dose optimization in both experimental and clinical applications. Clinically, this concept is reinforced by recent pilot trials and case series in soleus muscle injury, where ultrasound-guided PE combined with exercise produced better outcomes when higher intensity protocols were used, despite identical charge [9,10].

To our knowledge, this was the first study to objectively quantify structural changes in cadaveric muscle tissue in response to graded PE intensities using standardized quantitative ultrasound. This complements existing clinical research in chronic muscle pathology, such as the randomized trial by De-la-Cruz-Torres et al., which demonstrated that ultrasound-guided PE combined with eccentric training led to greater improvements in pain and function in dancers with chronic soleus injuries compared to isolated interventions [10].

Our findings complement this body of work by providing measurable, image-based metrics and reinforcing the concept that PE dosage generates reproducible, dose-dependent effects on muscle tissue. A pivotal contribution of this study was the development of the variable Muscle_Electrolysis_Dose, a composite index derived through multiple linear regression that consolidated the most representative structural features—specifically A_Area, A_Number, and A_Perimeter. This variable exhibited a clear upward trajectory in response to increasing current intensities, confirming its utility as a robust, objective indicator of the muscle’s structural response to PE. Its main advantage lay in its multidimensional nature, integrating several image-derived features into a single score, thereby enhancing interpretability while minimizing individual variability. This approach marked a shift from conventional reliance on subjective or categorical sonographic interpretation to a more reproducible and quantitative methodology. Previous PE studies in muscle have largely focused on clinical symptoms such as pain, range of motion, or performance, with limited emphasis on real-time or post-treatment imaging biomarkers [9,10]. Our study addressed this gap by introducing a continuous variable capable of capturing subtle yet meaningful tissue alterations across a spectrum of galvanic intensities. This not only paves the way for more individualized PE dosing strategies but also aligns with emerging calls for greater standardization and objectivity in electrotherapy research. Future research could expand upon this by integrating our structural findings with functional interventions, as prior studies have shown that combining PE with exercise yields superior clinical results in chronic muscle injuries [10,18]. Furthermore, the incorporation of quantitative ultrasound as a therapeutic monitoring tool—rather than solely for diagnosis—represents an important evolution in the application of musculoskeletal ultrasound in interventional physiotherapy and rehabilitation. From a practical perspective, these findings offered initial guidance for clinical protocols involving PE in muscle. Specifically, intensities between 1 and 4 mA appeared to produce the most consistent structural effects while minimizing artifact-related limitations, suggesting this range as a potentially safe and effective therapeutic window for muscular applications. Furthermore, the use of quantitative ultrasound as a monitoring tool could help clinicians visualize and titrate the impact of galvanic current in real time, promoting more individualized and reproducible treatment strategies.

Despite the promising nature of these findings, our study had several limitations. The use of cadaveric tissue precluded the assessment of true biological regeneration, as no inflammatory or cellular responses could be observed. Another important limitation was the lack of detailed donor medical histories. Due to ethical and legal constraints, comorbidities such as diabetes, cancer, or neuromuscular conditions could not be assessed or excluded, which may have introduced variability in tissue responses. Additionally, the application time of one second per dose, while standardized for experimental consistency, did not mirror typical clinical durations. Finally, the echogenic changes observed at high intensities may have partly reflected imaging artifacts rather than additional structural impact. Future research should validate these findings in vivo, assess the correlation between ultrasound-detected changes and clinical outcomes, and explore the implementation of Muscle_Electrolysis_Dose as a biofeedback tool for real-time PE dosing.

While the results of this study support the feasibility of using quantitative ultrasound to guide galvanic current dosing in percutaneous electrolysis, clinical translation will require addressing several practical considerations. Ultrasound image acquisition and interpretation are inherently operator-dependent, and variability across devices and scanning protocols could influence reproducibility. To mitigate these limitations, future developments should focus on standardizing ultrasound acquisition protocols and integrating real-time analysis tools—such as AI-based segmentation and automated feature extraction—that reduce user dependency. These innovations could ultimately lead to the development of biofeedback-driven PE systems that tailor current dosage based on real-time image-based tissue response, enhancing precision and safety in clinical practice.

## 5. Conclusions

The application of galvanic current to muscle tissue induces structural changes that are detectable through quantitative ultrasound imaging. A clear dose–response relationship was identified in key parameters such as A_Number, A_Area, A_Perimeter, A_Homogeneity, and A_Convexity, confirming that PE produces measurable alterations in muscle architecture.

Moreover, a novel predictive variable—Muscle_Electrolysis_Dose—was developed through a multiple linear regression model integrating A_Area, A_Number, and A_Perimeter, explaining 66.7% of the variance in the applied dose. This composite index represents a step forward toward more precise and personalized dosing by summarizing the tissue response into a single quantifiable metric. The segmentation of current intensities revealed that structural effects began to appear clearly from 1 mA, with a progressive stabilization beyond 4 mA, suggesting the presence of a physiological threshold in muscle tissue response.

This study introduced an innovative approach to PE dosing, incorporating quantitative ultrasound as an objective criterion to tailor the applied intensity. These findings lay the groundwork for future research exploring its clinical applicability, ultimately promoting more effective and physiologically informed treatment strategies.

## Figures and Tables

**Table 1:** Quantitative ultrasound variables analyzed to evaluate the structural effects of percutaneous electrolysis on muscle tissue.

Variable	Description	Interpretation
A_Number	Number of affected areas detected in the ultrasound image after current application.	Reflects the appearance of visible structural alterations induced by electrolysis.
A_Area	Total area of the altered region after the intervention.	Assesses the magnitude of the effect induced by electrolysis.
A_Perimeter	Perimeter of the affected region, providing information about the boundaries of tissue alteration.	Indicates the extent of structural effects induced by electrolysis.
A_Convexity	Ratio between the perimeter of the altered region and the perimeter of its convex hull.	Quantifies the geometric regularity of the affected area; lower values suggest greater irregularity and tissue disruption.
A_Homogeneity:	Measurement of echogenicity uniformity in the image.	A reduction suggests increased heterogeneity in muscle structure induced by electrolysis.
A_Contrast	Represents the level of contrast between the treated region and the healthy tissue.	An increase indicates greater differentiation in the ultrasound signal induced by electrolysis.
A_ASM	Texture parameter reflecting pixel organization regularity in the ultrasound image.	A decrease suggests greater disruption of the muscle structure induced by electrolysis.
B_GLCM and B_GLDS	Set of variables derived from texture co-occurrence matrices.	Assesses the structural complexity of the tissue following electrolysis.

ASM: Angular Second Moment; GLCM: Gray-Level Co-occurrence; GLDS: Gray-Level Difference Statistics.

**Table 2:** Spearman’s correlation analysis between galvanic current dose and the evaluated ultrasound parameters.

Variable	Spearman’s Rho	ρ-Value
A_Number	0.762	<0.001
A_Area	0.877	<0.001
A_Perimeter	0.873	<0.001
A_Convexity	−0.696	<0.001
A_Homogeneity	−0.828	<0.001
A_Contrast	0.794	<0.001
A_ASM	−0.101	0.602
B_GLCM_Contrast	0.173	0.370
B_GLCM_SumAverage	0.245	0.201
B_GLCM_SoSVariance	0.470	0.010
B_GLCM_DVariance	−0.500	0.006
B_GLCM_Correlation	0.558	0.002
B_GLCM_IDMoment	−0.346	0.066
B_GLDS_Homogeneity	0.379	0.043
B_GLDS_Contrast	0.368	0.050
B_GLDS_ASM	0.368	0.049
B_GLDS_Entopy	−0.383	0.040
B_GLDS_Mean	0.394	0.035
B_haar_mean	0.243	0.203
B_haar_variance	0.475	0.009

ASM: Angular Second Moment; GLCM: Gray-Level Co-occurrence; GLDS: Gray-Level Difference Statistics.

**Table 3:** Results of the linear regression analysis between galvanic current dose and the evaluated ultrasound variables.

Variable	R	R^2^	Standard Error	F	*p*-Value (ANOVA)	Beta	B (Dose Coefficient)	IC 95% (Lower–Upper)
A_Number	0.682	0.465	0.71475	23.488	<0.001	0.682	0.197	0.113–0.280
A_Area	0.734	0.538	0.01815	31.475	<0.001	0.734	0.006	0.004–0.008
A_Perimeter	0.793	0.629	0.39844	45.834	<0.001	0.793	0.153	0.107–0.200
A_Convexity	0.608	0.37	0.14995	12.338	0.002	−0.608	−0.035	−0.056–−0.014
A_Homogeneity	0.768	0.59	0.05896	38.866	<0.001	−0.768	−0.021	−0.028–−0.014
A_Contrast	0.724	0.524	87.72289	29.735	<0.001	0.724	27.151	16.934–37.367
B_GLCM_SoSVariance	0.487	0.237	196.93049	8.403	0.007	0.487	32.401	9.467–55.335
B_GLCM_DVariance	0.501	0.251	2 × 10^−5^	9.026	0.006	−0.501	−3.307 × 10^−6^	0.000–0.000
B_GLCM_Correlation	0.479	0.23	0.00895	8.057	0.009	0.479	0.001	0.000–0.002
B_GLDS_Homogeneity	0.322	0.104	1972.40533	3.118	0.089	0.322	197.685	−32.019–427.390
B_GLDS_Contrast	0.346	0.12	1,912,323.6	3.682	0.066	0.346	208,274.396	−14,433.070–430,981.862
B_GLDS_ASM	0.292	0.085	64,921,179.4	2.524	0.124	0.292	5,854,154.651	−1,706,506.81–13,414,816.1
B_GLDS_Entropy	0.344	0.118	103,069.017	3.616	0.068	−0.344	−11,124.839	−23,128.163–878.485
B_GLDS_Mean	0.362	0.131	105,617.858	4.072	0.054	0.362	12,097.469	−202.691–24,397.629
B_haar_variance	0.487	0.237	771.58943	8.398	0.007	0.487	126.916	37.058–216.775

ASM: Angular Second Moment; GLCM: Gray-Level Co-occurrence; GLDS: Gray-Level Difference Statistics.

**Table 4:** Results of the multiple linear regression analysis.

Variable	B (Coef)	Standard Error	Beta	t	Sig (*p*)	CI 95% Lower Bound	CI 95% Upper Bound
Constant	0.542	1.099	—	0.493	0.628	−1.759	2.843
A_Area	−316.88	109.090	−2.613	−2.905	0.009	−545.209	−88.554
A_Number	−2.678	1.146	−0.656	−2.336	0.031	−5.077	−0.279
A_Perimeter	20.284	5.506	3.826	3.684	0.002	8.761	31.808

**Table 5:** Kruskal–Wallis test results for evaluating differences across dose ranges (low: 0–1 mA, medium: 1–6 mA, and high: >6 mA) in the selected ultrasound-based variables.

Variable	N	Test Statistics	Degrees of Freedom	Asymptotic Significance (Two-Tailed)	Low vs. Medium (*p*)	Low vs. High (*p*)	Medium vs. High (*p*)
A_Number	29	16.94	2	<0.001	0.002	<0.001	0.077
A_Area	29	22.426	2	<0.001	<0.001	<0.001	0.013
A_Perimeter	29	22.053	2	<0.001	<0.001	<0.001	0.006
A_Convexity	29	10.771	2	0.005	0.050	0.003	0.041
A_Homogeneity	29	21.271	2	<0.001	<0.001	<0.001	0.026
A_Contrast	29	19.901	2	<0.001	<0.001	<0.001	0.016
B_GLCM_SoSVariance	29	8.588	2	0.014	0.020	0.008	0.929
B_GLCM_DVariance	29	6.958	2	0.031	0.024	0.032	0.534
B_GLCM_Correlation	29	11.357	2	0.003	0.020	0.002	0.248
B_haar_variance	29	8.408	2	0.015	0.024	0.008	0.790
UZ_eDosis	29	21.125	2	<0.001	0.003	<0.001	<0.001

## Data Availability

The data presented in this study are available on request from the corresponding author.
